# Mini-Review: Bioactivities of Bacterial Cell Envelopes in the Central Nervous System

**DOI:** 10.3389/fcimb.2020.588378

**Published:** 2020-10-26

**Authors:** William J. MacCain, Elaine I. Tuomanen

**Affiliations:** Department of Infectious Diseases, St. Jude Children's Research Hospital, Memphis, TN, United States

**Keywords:** meningitis, PAMP, pattern recognition receptor, peptidoglycan, neurodevelopment

## Abstract

During acute bacterial meningitis, recognition of the bacterial envelope by immune cells of the central nervous system (CNS) generates a robust response that is essential to clear bacteria. This response is further amplified during treatment when lytic antibiotics, required for cure, also generate a burst of highly inflammatory cell envelope debris. Different peptidoglycan (PG) subcomponents interact with neurons, glia, and the blood brain barrier resulting in the entire symptom complex of meningitis. Recently, this CNS-cell envelope signaling axis has been extended to non-inflammatory recognition of cell wall components circulating from endogenous bacteria to the brain resulting in both benefit and chronic damage. This review will describe the molecular details of a broad array of cell envelope-induced responses in the CNS and what current strategies can be implemented to improve clinical outcome.

## Introduction

The bacterial cell envelope is one of the most powerful pathogen associated molecular patterns (PAMP) recognized by pattern recognition receptors (PRR) of the human immune system. In the central nervous system (CNS), detection of the cell envelope is critical to the course of bacterial meningitis, one of the most dreaded infectious diseases. *Streptococcus pneumoniae, Neisseria meningitidis*, and *Hemophilus influenzae* are the three classical pathogens associated with bacterial meningitis (McGill et al., 2016). PG from the cell envelopes elicit signs and symptoms ranging from drowsiness, fever, headache and neck stiffness to severe intracranial pressure and widespread neuronal death with permanent sequelae and an overall fatality rate between 20 and 30% (van de Beek et al., 2002; Christie et al., 2011). Patients are treated with bacteriolytic antibiotics that are required for cure but also cause the rapid release of highly reactive bacterial debris (Tuomanen et al., 1985a). These PG fragments and cell envelope polymers persist and continue to elicit the influx of leukocytes, brain edema, inflammatory cytokines, and neuronal death, causing further neurological damage.

Interactions between cell wall-PAMPs and PRRs go beyond acute infection. Throughout life, PG fragments are released from the microbiome and circulate in serum (Clarke et al., 2010; Molinaro et al., 2019). Upon access to the CNS these fragments can influence neuronal development (Humann et al., 2016) or contribute to neurodegeneration (Laman et al., 2020). Here, we will describe the molecular interactions between a broad array of cell envelope fragments with specific receptors in the CNS that contribute to a spectrum of responses from CNS homeostasis to acute or chronic inflammation.

## Bacterial Envelope PAMPs

**Figure 1:**
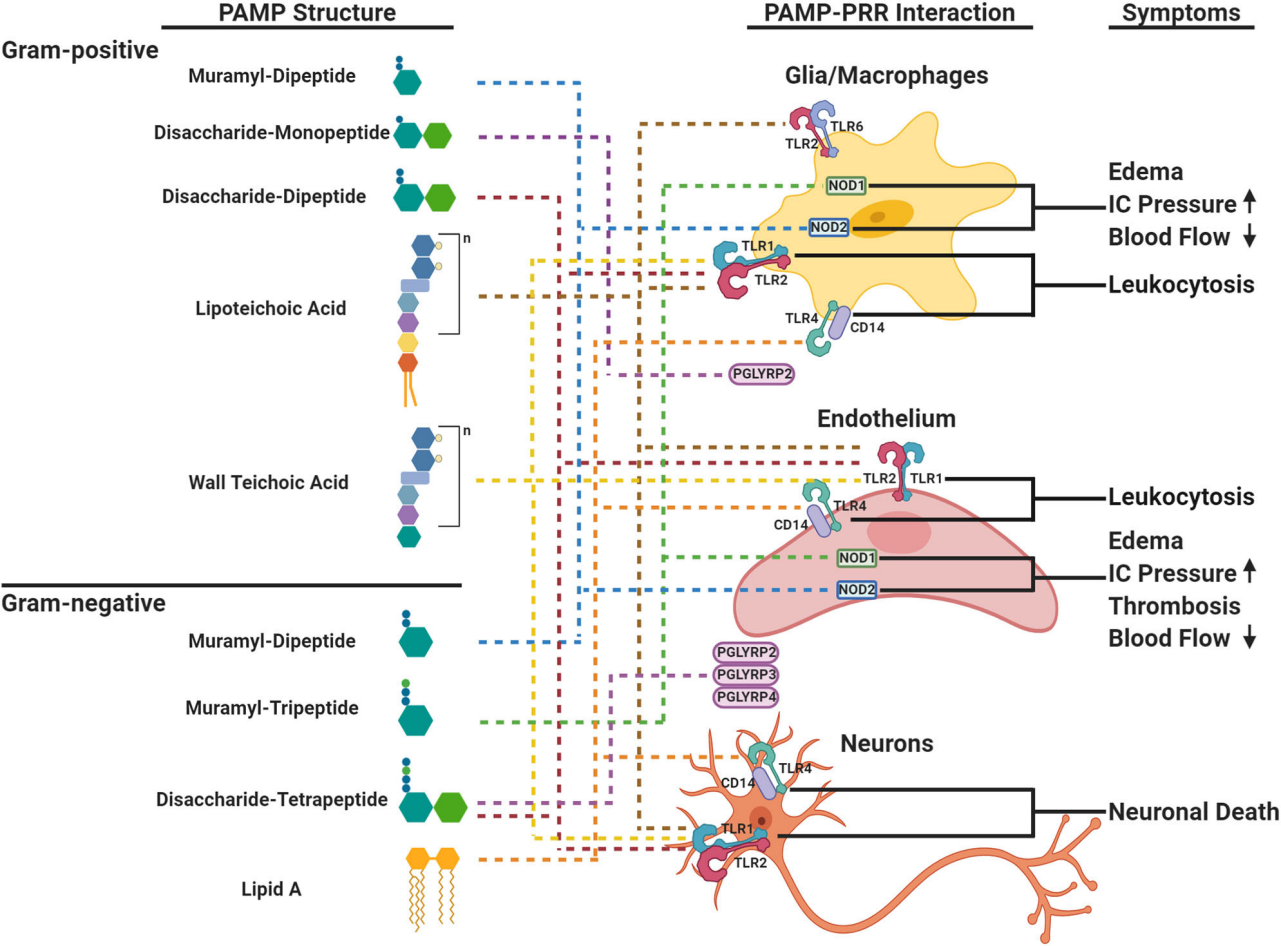
Schematic representation of cell envelope bioactivities during acute bacterial meningitis. The cell envelope is composed of a peptidoglycan (PG) backbone that is decorated and intertwined with wall teichoic acid (TA) or lipoteichoic acid (LTA) in Gram-positive (top panel) and PG and the lipid A components of lipopolysaccharide (LPS) in Gram-negative bacteria (bottom panel), respectively. PAMPs within PG, LTA, or LPS are ligands to PRRs (TLRs, NODs, PGLYRPs shown on cells in right panel) in the brain that elicit distinct major symptoms (right panel) during acute bacterial meningitis. Each component is linked to its cognate PRR by dotted lines. TLR, Toll-like Receptor; NOD, Nucleotide-binding Oligomerization Domain-like Receptor; PGLYRP, Peptidoglycan Recognition Proteins; IC, Intracranial.

For both Gram positive and negative bacteria, the network of PG is most notable for protecting bacteria against osmotic forces (Silhavy et al., 2010) and maintaining cell shape (Young, 2006). The PG is a highly conserved repeating disaccharide of N-acetylglucosamine (GlcNac) and N-acetylmuramic acid (MurNac) that forms long chains interconnected by cross-linked peptides of varied length (Figure 1). In Gram-positive bacteria, the entirety of the cell wall is composed of a thick ≈20-80 nm layer of PG that is covalently linked to wall teichoic acids (TA) and interlaced with membrane-bound lipoteichoic acids (LTA) (Skov Sorensen et al., 1988; Tomasz, 2000). The cell envelope of Gram-negative bacteria is composed of a thin 5–10 nm layer of PG covalently linked to lipoproteins and commonly co-mingled with outer membrane-bound lipopolysaccharide (LPS) (Matias and Beveridge, 2007). The bioactivities of PG, LTA, and LPS are unique for each pathogen as they are affected by small molecular adducts to the glycan backbone, variations in TAs and LPS, and by the detailed variability of the cross-links between invariant stem peptides. For this review, we will focus on the PG, LTA, and LPS as the main sources of bacteria-associated inflammation, and going forward, refer to these three structures as components of the cell envelope. When we refer to the PG, TA, or muropeptides, we will use the term cell wall.

Within the cell wall structure, LTA and LPS, the innate immune system recognizes PAMPs through PRRs that can signal downstream events to combat infection. The highly conserved PG presents PAMPs derived from fragments of muropeptides that are variations of GlcNac-MurNac- pentapeptides (Figure 1). PRRs distinguish between variations of the third residue of the pentapeptide: meso-diaminopmelic acid (m-DAP) for Gram-negatives and lysine for Gram-positives. The diversity of muropeptide structures is further generated by autolysins, such as peptidases, amidases, and glycosylases. There can be as many as 80 different muropeptides in a bacteria's PG network (Glauner, 1988; Burroughs et al., 1993b; Bui et al., 2012), each with variable ability to invoke a host immune response.

In addition to muropeptides, LPS, LTA and TA are recognized by PRRs (Figure 1). LPS has a tripartite structure: a lipid A anchor, core oligosaccharide, and O-antigen polysaccharide. Different bacteria produce structurally different LPSs that vary in immunostimulatory potency based on phosphorylation, length of oligosaccharides, and composition (Pridmore et al., 2003; Albiger et al., 2007). TAs are linked to the PG MurNac residue and are formed by repeats of ribitol phosphate or glycerol phosphate further decorated by D-alanine and/or sugar residues (Tomasz, 2000; Kang et al., 2016). Most Gram-positive organisms have starkly distinct LTA and TA. However, *S. pneumoniae* is unique because TA and LTA are not only identical, but also both are decorated with phosphorylcholine (ChoP) (Briles and Tomasz, 1973). The ChoP adduct, a decoration shared by the three common meningeal pathogens on their surfaces, mimics the chemokine platelet-activating factor (PAF) and thereby mediates binding of bacteria to the PAF receptor. This actively promotes trafficking of bacteria and cell surface fragments across epithelia and endothelia, including the blood brain barrier (BBB) (Cundell et al., 1995; Clark and Weiser, 2013). Localizing ChoP to TA makes the pneumococcal cell wall a unique PAF receptor ligand and adds to its distribution during infection and its bioactivities (Cundell et al., 1995).

## PRRs For Cell Envelope Components

There is a wide range of PRR pathways that can be triggered by bacterial envelope components to induce a proinflammatory response within the CNS. The families of PRRs are broken down into Toll-Like receptors (TLRs), NOD-like receptors (NLRs), PG recognition proteins (PGLYRPs), and accessory co-receptors such as lipopolysaccharide-binding proteins (LBP, MD-2, and CD-14). While each PRR recognizes a subset of PAMPs, there is considerable plasticity in ligand recognition, which varies by body site and by the presence of various co-receptors (Li et al., 2013). Extracellular TLRs 1,2, 4, and 6 signal when ligands induce the formation of homo- or heterodimers. TLR2 generally recognizes Gram-positive components such as LTA, di- and tri-acylated lipoproteins and PG fragments. Discrimination of subtle differences in lipids of lipoproteins is conferred by dimerization with TLR1 or TLR6 and the transfer of ligands from the co-receptors CD14, CD36, or LBP (Yoshimura et al., 1999; Han et al., 2003; Hoebe et al., 2005; Akira et al., 2006; Jin et al., 2007). The crystal structures of TLR2/1 and 2/6 indicate direct binding to their preferred lipopeptide ligands (Jin et al., 2007). In contrast to TLR2, TLR4 is a homodimer that recognizes Gram-negative components such as LPS, usually in the presence of the co-receptors CD14, MD-2, and LBP (Medzhitov et al., 1997). This division between TLR2 and 4 is not absolute as Gram-negative muropeptides have been reported to bind TLR2 (Asong et al., 2009) and TLR4 co-receptors have been implicated in binding PG fragments and LTA, thereby expanding classical TLR2 ligands to TLR4 and vice versa (Dziarski et al., 1998; Weber et al., 2003a). The final result of TLR2 or 4 signaling is activation of NF-κB and production of inflammatory cytokines.

The cytoplasmic NOD receptors recognize muropeptides within the host cell. NOD1 recognizes the m-DAP portion of Gram-negative muropeptides (Girardin et al., 2003a), and NOD2 recognizes muropeptides with both m-DAP and lysine. The MurNac residue is dispensable for NOD1 activation, but is required for NOD2 activation and can be linked to either a dipeptide or tripeptide moiety (Girardin et al., 2003b). Unlike the TLRs, NLRs can trigger the expression of inflammatory and antimicrobial genes through the NF-κB and MAPK pathways (Mukherjee et al., 2019).

There are four PGLYRPs in human serum, and they each have a different high affinity for LPS, LTA, and PG muropeptides (Royet and Dziarski, 2007). Similar to the NODs, the PGLYRPs differentiate between muropeptides based on the third amino residue being m-DAP or lysine (Kumar et al., 2005). Interestingly, PGLYRP2 is the only PGLYRP with amidase activity that cleaves PG (Wang et al., 2003).

## Cell Envelope Modification to Avoid PRR Detection

Pathogenic and commensal bacteria modify their PG to evade detection by the innate immune system. There are two strategies: modification of the glycan backbone (N-deacetylation, N-glycosylation, and acetylation) and modification to the stem peptides. For example, *S. pneumoniae* encodes Adr, O-acetyl transferase, and PgdA, acetylglucosamine deacetylase, that modify the C-6 position of the MurNac and the C-2 position of GlcNac, respectively (Vollmer and Tomasz, 2000; Crisostomo et al., 2006). These modifications prevent degradation by lysozyme and reduce recognition by NOD1 (Boneca et al., 2007). Certain bacteria contain L-ornithine instead of m-DAP in their stem peptides to avoid detection by the NLR family (Girardin et al., 2003b).

Modifications to the LPS, TA, and LTA contribute to immune dampening and avoidance. Meningococcus and *H. influenzae* shorten their O-antigens in LPS to dampen reactivity. To decrease susceptibility to antimicrobial peptides, ethanolamine, and aminoarabinose can be added to LPS (Guo et al., 1998) and D-alanyl can be added to TAs (Collins et al., 2002).

## Cell Envelope Release: Antibiotics vs. Normal Growth

Cell envelope components are released into the cerebrospinal fluid (CSF) gradually during growth or rapidly during antibiotic-induced autolysis (Tuomanen et al., 1985a; Arditi et al., 1989; Woodhams et al., 2013; Gonzalez-Santana and Diaz Heijtz, 2020). All three meningeal pathogens are autolytic and therefore undergo rapid cell envelope release during stationary phase and during antibiotic therapy. β-lactam antibiotics, the first line of therapy for meningitis, inhibit PG synthetic enzymes and activate autolysins that destabilize the cell wall network. The result is the release of an array of constituent muropeptides, TA, LTA, and LPS, each with different inflammatory bioactivities (Tuomanen et al., 1985b; Arditi et al., 1989; Burroughs et al., 1993a; Eng et al., 1993; Schneider et al., 1999; Woodhams et al., 2013). It is well-recognized that higher concentrations of PG and LPS in the CSF correlate with poor clinical outcome during meningitis (Arditi et al., 1989; Brandtzaeg et al., 1992; Schneider et al., 1999; Grandgirard et al., 2010).

Given that bactericidal antibiotics are required for curing meningitis and these drugs necessarily release a burst of cell envelope during autolysis, efforts to decrease the resultant neurological damage has focused on dampening the rapid increase in inflammation during the initial doses of antibiotics. In animal models and clinical trials, dexamethasone or non-steroidals, co-administered with the first dose of antibiotics, decrease inflammation and improve outcome (Tuomanen et al., 1987; Odio et al., 1991). Specifically, these measures reduce hearing loss, neurological sequelae and significantly reduce overall mortality (Brouwer et al., 2015; Rayanakorn et al., 2020).

## Relative Specific Activities of Cell Envelope Components

The cardinal signs of meningitis in the CSF are a high neutrophil count, protein accumulation associated with BBB permeability, increased intracranial pressure due to brain edema within the closed space of the skull, and low glucose as cerebral metabolism is compromised. Except for low glucose, all of these clinical signs can be recapitulated by infusion of specific cell envelope components into the CSF in animal models indicating that the cell envelope can be considered a library of bioactive PAMPs that together add up to the constellation of pathophysiology of meningitis (Figure 1). The specific activity of key bacterial surface components for inciting CSF inflammatory changes can be calculated (Table 1). The onset of bacterial seeding of the CSF from blood occurs at ~10e5 bacteria/ml and rises to 10e7 bacteria/ml at symptomatic disease (Arditi et al., 1989). The cardinal signs of meningitis can be induced by intact *Hemophilus* or pneumococcus at ~10e5 bacteria which represents 1 ng of LPS/PG or 10 ng LTA/PG-TA: protein influx at 2 h, leukocytosis at 4 h, and brain edema at 6 h post intracisternal injection (Tuomanen et al., 1985b; Burroughs et al., 1993a). More complex components sustain equal or greater activity at 24 h. Yet, each surface component exhibits a different dose response relationship depending on the pathophysiological sign (Table 1). For *Hemophilus*, LPS strongly invokes leukocytosis (threshold 10 ng/ml) while PG powerfully induces brain edema (threshold 1 ng/ml). The activity of PG is not affected by the presence of proteins, increases 10 fold if solubilized (autolysis product), and can vary by 2 fold depending on the stem peptide composition (Burroughs et al., 1992, 1993a). In contrast, for pneumococcus, LTA and TA specifically invoke leukocytosis (threshold 500 ng/ml) while multimeric disaccharide stem peptides (autolysis product) powerfully induce both leukocytosis and brain edema (threshold 100 ng/ml) (Tuomanen et al., 1985b; Tomasz and Saukkonen, 1989; Weber et al., 2003b). The disaccharide tetrapeptide, the most common PG building block, is most active for brain edema rather than leukocytosis. The high specific activity of free stem peptides for brain edema (threshold 1 ng/ml) is not due to any one super peptide. Specific bioactivities of the 1,6-anhydromuramyl peptide found at the end of glycan chains includes induction of slow-wave sleep (Krueger et al., 1984) and mitochondrial toxicity in ciliated cells, such as in the choroid plexus (Cookson et al., 1989). In summary, many subcomponents of cell envelopes are bioactive in the range of 10e5–10e7 bacterial equivalents, values found routinely in CSF during meningitis.

**Table 1:** Threshold (ng/ml) for CNS bioactivity of bacterial components.

**Component**	**Leukocytosis**	**Edema**
**Gram negative**		
LPS + PG	5	5
LPS	10	100
PG	100	1
**Gram positive**		
LTA + PG	100	10
LTA	500	–
PG-TA w/stems	20	100
PG	100	–
Stem peptides	10,000	1
TA free	500	10,000

*Compiled from: (Tuomanen et al., 1985a,b; Tomasz and Saukkonen, 1989; Burroughs et al., 1992, 1993a,b; Weber et al., 2003a,b)*.

## Pathophysiological Host-Cell Envelope Mediated Inflammatory Responses

PAMPs are detected and interpreted by PRRs on key CNS cells: cerebral capillary endothelial cells of the BBB, glia (microglia, astrocytes, and oligodendrocyte cells) as the resident equivalent of leukocytes, neurons, and incoming peripheral blood neutrophils. All of these cells express the full array of PRRs: TLRs, NODs, PGRPs, but the results of their activation are cell specific and ligand specific (Bsibsi et al., 2002).

Glia serve as sentinels and are essential in the early innate immune recognition of LPS, LTA, and muramyl-peptides via their cognate receptors: TLR4, TLR2, and NODs, respectively (Bsibsi et al., 2002; Olson and Miller, 2004; Liu et al., 2010). The TLR signaling pathways act through MyD88 to activate NF-κB resulting in production of proinflammatory cytokines such as TNF-α, IL-1, IL-6, and IL-8. IL-1 and TNF-α contribute to the production of vasoactive nitric oxide, which in turn increases intracranial pressure and reduces oxygen uptake (Suschek et al., 1993; Tureen, 1995). It is also important to recognize that TLRs are not always proinflammatory. In fact, cell wall induced TLR2-NOD2-RIPK2 signaling leads to the production of the anti-inflammatory cytokine IL-10, which serves to shut down inflammation as infection resolves (Moreira et al., 2008).

Cerebral endothelial cells form tight junctional complexes that seal the BBB as a barrier from blood. Some small PG fragments, such as dipeptide D-Glu-mDAP and muramyl-dipeptide, are internalized and activate NF-κB through the NLR pathway amplifying the production of proinflammatory cytokines, chemokines (CCL2/3, CXCL1/8, and MIP-2), and prostaglandins (PGE2). These signaling molecules lead to the disruption of the BBB in conjunction with the recruitment of peripheral white blood cells. In bacterial meningitis, neutrophils comprise >90% of infiltrating leukocytes, and cause vasculitis, hemorrhage, and edema (Polfliet et al., 2001; Koedel et al., 2009). Prolonged leukocytosis increases the release of matrix metalloproteinases that degrade collagen IV and fibronectin, which are crucial components of the subendothelial basal lamina furthering the breakdown of the BBB (Lukes et al., 1999). Endothelial-derived tissue type plasminogen activator (tPA) leaks into the CSF, triggering thrombosis and a decrease in blood flow (Winkler et al., 2002). The presence of thrombin activates endothelial cells to produce PAF causing edema (Zimmerman et al., 1985).

Human neurons express TLR1, TLR2, TLR4, and CD14 for the recognition of LTA and the Lipid A portion of LPS (Acosta and Davies, 2008; Dzamko et al., 2017). Neuronal death through caspase-3 dependent apoptosis is driven by PG, LTA and LPS through proinflammatory cytokines (Mitchell et al., 2004). Neurons in the hippocampus, prefrontal cortex, and cerebellum highly express PGLYRP2 and NOD1 for the recognition and distinction of muropeptides from both Gram-positive and Gram-negative bacteria (Arentsen et al., 2017). PGLYRP2 binds to the bacterial cell wall to cleave the stem peptide, and activation of NOD1 causes the production of proinflammatory cytokines (IL-1β, TNF-α, IL-6) (Acarin et al., 2000).

## Host-Cell Envelope Interactions in CNS Development and Neurodegeneration

Although cell envelope PAMPs classically stimulate an acute inflammatory response, there is increasing recognition of their roles in modulating an array of non-inflammatory outcomes in the CNS, ranging from embryonic development to neurodegeneration. Muropeptides from the gut microbiome normally circulate constantly in serum (Clarke et al., 2010). Thus, there is ample opportunity for muropeptide/PRR interactions to occur without frank infection of the CNS. Furthermore, the gut microbiota communicates directly with the brain through the vagus nerve (Fulling et al., 2019), modulating the formation of the BBB (Braniste et al., 2014), and maturation of microglia (Erny et al., 2015).

Evidence implicates interactions of PRRs and cell envelope components in chronic neuroinflammation associated with neurodegeneration and behavioral abnormalities (Laman et al., 2020). Chronic, tonic signals from the microbiome are sensed by neurons and may exacerbate neurodegeneration. LPS can be found at high concentrations in the hippocampus and superior temporal lobe neocortex during Alzheimer's disease (Zhao et al., 2017). Loss of *nod1* and *nod2* severely decreases serotonin levels in the hippocampus and brainstem, which is associated with cognitive impairment and depressive-like behaviors (Pusceddu et al., 2019). Thus, bioactivities encoded in cell envelope components may incite a greater array of pathological processes than just simply acute inflammation during infection.

PRRs are expressed early in fetal development and are found on virtually all CNS cell types throughout life (Okun et al., 2010). Some PG-TA components generated during treatment of maternal infection are capable of crossing the placenta and entering the fetal brain where they alter neurodevelopment (Humann et al., 2016). Early in fetal development, interactions of PG-TA with neuronal TLRs silently induce overproliferation of neuronal progenitors leading to an abnormal increase in cell number in the neocortex in murine models (Humann et al., 2016). This aberrant architecture is permanent and associated with behavioral abnormalities after birth. Absence of PGLYRP2 is associated with altered expression of the brain-derived neurotrophic factor altering formation and regulation of neural circuits (Park and Poo, 2013; Arentsen et al., 2017; Eagleson et al., 2017). Thus, both the presence of excess PG-TA during early windows of development or the absence of PG sensors can lead to abnormal CNS development.

## Conclusion

The bacterial cell envelope is a highly bioactive library of molecules driving the course of acute and chronic inflammation and perhaps emerging as modifiers of normal development. Each pathogen presents its own array of PRR ligands and thus builds its own pattern of disease. The CNS has a complete and diverse array of innate sensors to engage cell envelope subcomponents and respond with induction of signaling. It appears that timing is key to damage or benefit to outcome. Bursts of PRR-induced signaling are generally damaging while the low level, constant signaling produced by microbiome components, may serve as an ever present source of communication from peripheral organs to the CNS.

## Author Contributions

WM wrote the initial draft and ET reviewed the draft and wrote the final manuscript.

## Conflict of Interest

The authors declare that the research was conducted in the absence of any commercial or financial relationships that could be construed as a potential conflict of interest.
